# Prophylactic Vaccination and Intratumoral Boost with HER2-Expressing Oncolytic Herpes Simplex Virus Induces Robust and Persistent Immune Response against HER2-Positive Tumor Cells

**DOI:** 10.3390/vaccines11121805

**Published:** 2023-12-02

**Authors:** Zahid Delwar, Olga Tatsiy, Dmitry V. Chouljenko, I-Fang Lee, Guoyu Liu, Xiaohu Liu, Luke Bu, Jun Ding, Manu Singh, Yanal M. Murad, William Wei-Guo Jia

**Affiliations:** Virogin Biotech Canada Ltd., Vancouver, BC V6V 3A4, Canada; zdelwar@virogin.com (Z.D.);

**Keywords:** oncolytic virus, herpes simplex virus, cancer vaccine, HER2

## Abstract

The development of effective cancer vaccines remains a significant challenge due to immune tolerance and limited clinical benefits. Oncolytic herpes simplex virus type 1 (oHSV-1) has shown promise as a cancer therapy, but efficacy is often limited in advanced cancers. In this study, we constructed and characterized a novel oHSV-1 virus (VG22401) expressing the human epidermal growth factor receptor 2 (HER2), a transmembrane glycoprotein overexpressed in many carcinomas. VG22401 exhibited efficient replication and HER2 payload expression in both human and mouse colorectal cancer cells. Mice immunized with VG22401 showed significant binding of serum anti-HER2 antibodies to HER2-expressing tumor cells, inducing antibody-dependent cell-mediated cytotoxicity (ADCC) and complement-dependent cytotoxicity (CDC). Furthermore, mice primed with VG22401 and intratumorally boosted with the same virus showed enhanced antitumor efficacy in a bilateral syngeneic HER2(+) tumor model, compared to HER2-null backbone virus. This effect was accompanied by the induction of anti-HER2 T cell responses. Our findings suggest that peripheral priming with HER2-expressing oHSV-1 followed by an intratumoral boost with the same virus can significantly enhance antitumor immunity and efficacy, presenting a promising strategy for cancer immunotherapy.

## 1. Introduction

Cancer is one of the leading causes of death worldwide, and traditional therapies such as surgery, chemotherapy, radiation therapy, hormonal therapies, and targeted therapies provide inadequate durable responses in patients with advanced cancers [1]. Recently, genetically modified HSV-1 vectors have emerged as promising therapeutic agents to treat various cancers due to their ability to selectively replicate in tumor cells, leading to tumor cell lysis while sparing non-malignant cells [2]. Oncolytic herpes simplex virus type 1 (oHSV-1) has been explored for more than a decade in laboratories and clinical trials for the treatment of many types of cancer [3]. In 2015, the herpes-virus-based oncolytic virus (oHSV-1) T-VEC, an oncolytic HSV-1 carrying GM-CSF, became the first oncolytic virus (OV) to be approved by the FDA for treating melanoma [4]. Viral gene mutations or deletions of one or multiple viral genes are the most common approaches to making the HSV-1 tumor specific [5]. T-VEC was attenuated by deletion of the neurovirulence gene ICP34.5, which significantly compromised its ability to replicate in normal cells while enabling selective replication in tumor cells that lack protein kinase R (PKR) activity [6]. Recently, another HSV-1-based oncolytic virotherapy, Delytact, was approved in Japan for treating malignant glioma [7].

In recent years, cancer immunotherapeutic approaches have emerged as promising strategies for cancer treatment, with PD-1 and CTLA-4 antibodies successfully used as checkpoint inhibitors to treat several types of cancer [8]. Nevertheless, only a fraction of cancer patients show a complete response after treatment [9]. Another frequently investigated cancer immunotherapeutic strategy involves peptide vaccines, which have emerged as a promising treatment option for patients with breast cancer, with significant advancements having been made in recent times. The mechanism of action of peptide vaccines typically involves three stages. Initially, the injected peptide is ingested by antigen-presenting cells (APCs). Next, CD8+ T cells recognize the APCs and produce antigen-specific cytotoxic T lymphocytes (CTLs). Finally, these CTLs identify and attack tumor cells expressing the antigen, releasing cytokines and perforin to dissolve them [10,11].

The development of several human cancers is closely linked to the human epidermal growth factor receptor (HER) family of receptors, which are responsible for controlling a variety of cellular functions such as growth, survival, and differentiation through a range of signal transduction pathways [12]. HER2 (human epidermal growth factor receptor 2), encoded by the ERBB2 gene situated on the long arm of human chromosome 17 (17q12), is a transmembrane glycoprotein that consists of 1255 amino acids with a molecular weight of 185 kD [13]. The initiation of HER2 signaling pathways causes the phosphorylation of tyrosine residues, which then activate downstream signaling pathways including Ras/MEK/ERK, JAK/STAT, and PI3K/AKT. Overexpression of HER2 is observed in various types of cancer. Additionally, it is important to note that HER2 overexpression is linked to a more aggressive form of the disease, a higher incidence of recurrence, and a shorter overall survival time [14].

While HER2 is classified as a tumor-associated antigen (TAA), pre-existing anti-HER2 immunity levels are typically too low to trigger an apparent therapeutic effect [15]. Thus, vaccines targeting HER2-related antigens need to overcome existing immune tolerance to generate a strong and durable immune response. The use of HER2/ERBB2-targeted vaccines has shown potential in the fight against breast cancer, with different HER2-based vaccines being developed over the years [16]. Recent studies have shown that a HER2-plasmid-based vaccine resulted in the production of HER2-specific type 1 T cells in the majority of patients with HER2-positive breast cancer [17].

Combining vaccines targeting HER2-related antigens with oncolytic herpes virus therapy can leverage the high immunogenicity of HSV vectors to facilitate strong tumor-localized inflammation and induce an in situ cancer vaccination effect [18,19,20]. Several cell types have been demonstrated to play a role in the innate immune response to HSV [21]. Natural killer (NK) cells are one of the most significant, contributing to anti-HSV immunity through cytokine production, recognition, and killing of virally infected cells [22]. Plasmacytoid dendritic cells (pDCs) also play a critical role in this process by producing type I interferon [23]. In addition, studies have demonstrated that CD8+ T cells are recruited to HSV lesions within 48 h after infection, significantly contributing to immune control and cytolysis [24].

In the present study, we tested a prime–boost vaccination regime utilizing oHSV-1 expressing HER2. The recombinant HSV used for vaccination (VG22401) was built on the TTDR oHSV-1 backbone VG201 that we have previously reported [25], and that is currently in phase I clinical trials. VG22401 used a tumor-specific CXCR4 promoter to drive the expression of the essential viral gene ICP27. We also employed microRNA regulation of ICP34.5 using microRNAs that were highly expressed in neuronal and normal cells but not in tumor cells [26,27]. All of the viral backbones also expressed IL-12, IL-15, and IL-15 receptor alpha (Ra) subunits [28]. We found that HER2-expressing oHSV-1 elicits strong anti-HER2 immunity when subcutaneously administered. Furthermore, we observed that pre-immunization with HER2-expressing oHSV-1 followed by intratumoral administration of the same virus can significantly boost its antitumor efficacy by facilitating a HER2-antigen-specific T lymphocyte response.

## 2. Material and Methods

### 2.1. Cell Lines and Reagents

CT26.WT (CT26) murine colon carcinoma, HCT 116 human colon carcinoma, LS 174T human colon adenocarcinoma, and African green monkey kidney (Vero) cells were obtained from the American Type Culture Collection (ATCC; Manassas, VA, USA). HCT 116 and Vero cells were grown in Dulbecco’s modified Eagle’s (DMEM) media, while CT26 and LS174T cells were grown in Roswell Park Memorial Institute 1640 (RPMI) media. Each medium was supplemented with filtered, heat-inactivated fetal bovine serum (FBS; Gibco-BRL, Grand Island, NY, USA) and 100 μg/mL Primocin (InvivoGen, San Diego, CA, USA) and incubated in a cell culture incubator at 37 °C and 5% CO^2^.

The CT26-HER2 (HER2-expressing mouse tumor) cells were generated by transducing CT26 mouse cells (ATCC, Manassas, VA, USA) with a lentivirus vector (Genecopoeia, Rockville, MD, USA, ULP-Z2866-Lv105-A00) encoding the cDNA for human erbB-2. RPMI medium supplemented with 10% fetal bovine serum (FBS; Thermo Fisher Scientific, Waltham, MA, USA) was used for cell maintenance and propagation. Following transduction, the cells were selected with 2 µg/mL of puromycin (Sigma-Aldrich, St. Louis, MO, USA); then, single clones were isolated, expanded, and tested by flow cytometry (Figure S1) for stable expression of erbB-2 using Anti-ErbB2/c-Neu (Ab-5), clone TA-1 (Sigma-Aldrich, St. Louis, MO, USA).

### 2.2. Recombinant Virus Construction

All of the recombinant HSV-1 viruses used in this study were constructed using standard lambda-Red-mediated bacterial artificial chromosome (BAC) recombineering techniques in *Escherichia coli* using HSV-1 strain 17 as the backbone. Mutant BACs were isolated using a HiSpeed Plasmid Midi Kit (Qiagen, Frederick, MD, USA), followed by virus recovery in Vero cells after transfection with Lipofectamine™ 2000 (ThermoFisher Scientific, Waltham, MA, USA). Restriction enzyme digestion combined with targeted sequencing of modified genomic regions was used to verify the genome integrity of all novel recombinant HSV-1.

The construction and characterization of a transcriptional and translational dual-regulated (TTDR) HSV-1 was published previously [25]. VG22401 was designed to incorporate key features of the TTDR platform, including transcriptional regulation of the HSV-1 gene transactivator ICP27 using the tumor-specific CXCR4 promoter. In addition, the translational regulation of a single copy of the major neurovirulence determinant ICP34.5 via the inclusion of miR-124 and miR-143 binding sites in the 3′-untranslated region of ICP34.5 was also used. The second copy of ICP34.5 was deleted along with one of the genomic repeat regions. Additionally, the 28 C-terminal amino acids of the HSV-1 gene encoding glycoprotein B (gB) in VG22401 were truncated to enhance fusogenicity. VG22401 was engineered to express a payload cassette consisting of IL12, IL15, and the IL15 alpha receptor subunit isoform 1, with each element separated by 2A peptides. The cytokine payload was inserted between viral genes UL3 and UL4, and its expression was controlled by a cytomegalovirus (CMV) promoter. VG22401 further encoded an expression cassette for the extracellular domain of human HER2 driven by the EF1a promoter. This was inserted between the HSV-1 genes US1 and US2. VG2062 was the backbone virus for VG22401 construction, sharing all features with VG22401 except for the HER2 expression cassette.

### 2.3. Cell Viability Assay

Cells were placed in a 96-well plate at a density of 5 × 10^3^ and allowed to settle overnight. Afterwards, they were exposed to either vehicle control, or to different viruses with varying MOIs. To assess cell viability after 3 days of treatment, a Cell Counting KIT 8 (CCK-8, Sigma-Aldrich, St. Louis, MO, USA) assay was performed in accordance with the manufacturer’s instructions. Briefly, the cells were incubated with a 10% CCK-8 solution for 2.5 h at 37 °C. Measurement of cell viability was then conducted at 450 nm using a plate reader SpectraMax iD3 (Molecular Devices, San Jose, CA, USA).

### 2.4. ELISA Assay to Detect Anti-HER2 and Anti-gD IgG in Mouse Serum

Serum was isolated from mouse blood by centrifugation (2000 rpm for 10 min) to evaluate the relative levels of anti-HER2 and anti-gD IgG antibodies. Serum was stored at −80 °C before analysis. The recombinant extracellular domain of human HER2/ErbB2 (aa 23–652, Acro Biosystems, Newark, DE, USA, #HE2-H5225) or glycoprotein D of the herpes simplex virus was coated on ELISA plates in 100 µL of DPBS (Thermo Fisher Scientific, Waltham, MA, USA, #14190-136) overnight at RT, at a concentration of 1 µg /mL. Plates were washed thrice with DPBS and blocked with Blocking Buffer (1× ELISA Diluent, Invitrogen, Waltham, MA, USA, #00-4202-56) for 2 h. Mouse serum samples were evaluated at seven serial dilutions of 1:5000. Plates were protected from light and incubated for 2 h at RT. Anti-mouse IgG (Goat) with HRP (PerkinElmer, Waltham, MA, USA, NEF822001EA) was used to detect mouse antibodies. After washing the plates three times, 100 μL of TMB (Thermo Fisher Scientific, Waltham, MA, USA, #00-4201-56) was added for 10 min. Within 15 min of adding the stop solution (sulfuric Acid 2.0 N, VWR, Radnor, PA, USA, BDH7500-1), absorbance of the wells at 450 nm and 570 nm was measured by SpectraMax iD3 (Molecular Devices, San Jose, CA, USA).

### 2.5. Quantification of the HER2 Payload

Cells were cultured in 24-well plates at 37 °C under a 5% CO_2_ atmosphere. After they reached 90% confluence, cells were infected with different viruses and incubated for 48 h. Infected cell supernatants were collected and stored at −80 °C. The levels of HER2 in the supernatant of infected cells were determined by ELISA using Human Total ErbB2/HER2 DuoSet IC ELISA (R&D systems, Minneapolis, MN, USA, DY1129B) according to the manufacturer’s instructions. Results were measured by SpectraMax iD3 (Molecular Devices, San Jose, CA, USA).

### 2.6. Splenocyte Isolation

Mouse splenocytes were obtained by pressing mice spleens through a 40 µm cell strainer (Corning, New York, NY, USA, 352340), followed by incubation with ACK lysis buffer (Thermo Fisher Scientific, Waltham, MA, USA, A10492-01). The isolated splenocytes were frozen in heat-inactivated fetal bovine serum (Thermo Fisher Scientific, Waltham, MA, USA, 12483-020) and 10% dimethyl sulfoxide (Sigma-Aldrich, St. Louis, MO, USA, D2650-100ML) using a “Mr. Frosty” freezing container (Sigma-Aldrich, St. Louis, MO, USA, C1562) at −80 °C overnight.

### 2.7. ELISpot Assay

Numbers of IFN-γ-producing splenocytes were evaluated using the Mouse IFN-γ (ALP) ELISpot Plus kit (Mabtech, Cincinnati, OH, USA, 3321-4APT) according to the manufacturer’s instructions. Briefly, splenocytes (250,000 per well) were stimulated with PepMix ErbB2_ECD (2 µg/mL) (JPT, Berlin, Germany, PM-ERB_ECD) for 24 h. After stimulation, cells were washed off, and the plates were incubated with biotin-conjugated anti-mouse IFN-γ mAb for 2 h, followed by incubation with Streptavidin–ALP for 1 h. Spots were visualized using BCIP/NBT-plus substrate solution. ELISpot results were analyzed using a BIOREADER 7000-F-z (BIOSYS, Karben, Germany).

### 2.8. Quantification of Viral Copies by qPCR

Cells were cultured in 24-well plates at 37 °C under a 5% CO_2_ atmosphere, with or without viral infection, for 48 h. DNA was isolated using the DNeasy Blood and Tissue Kit (Qiagen, #69582, Frederick, MD, USA) according to the manufacturer’s instructions. Viral copies were measured by qPCR using primers and a probe specific for the ICP27 gene of HSV-1, as described earlier [29]. To quantify the ICP27 copy number, a standard curve was generated using a plasmid containing a known quantity of the target gene. The specificity of the qPCR assay was validated, and no cross-reactivity was observed with either human or mouse genomic DNA.

### 2.9. Staining of Serum Anti-HER2 Antibody Binding to HER2-Expressing Tumor Cells

CT26-HER2 and HER2-negative parental CT26 cells were co-incubated with a 1:100 dilution of serum on ice for 30 min, and then washed twice with staining buffer. APC-conjugated anti-mouse IgG antibody (Thermo Fisher Scientific, Waltham, MA, USA, 1:100 dilution) was applied to the cells, followed by incubation on ice for 30 min. Serum antibody binding to HER2 expressed on the cell surface was measured by flow cytometry.

### 2.10. Antibody-Dependent Cell-Mediated Cytotoxicity Assay

Antibody-dependent cell-mediated cytotoxicity (ADCC) was measured against HER2-expressing CT26 cells (CT26-HER2) and HER2-negative parental CT26 cells using an mFcgRIV ADCC Reporter Bioassay (Promega, Madison, WI, USA, M1201). Target cells (7500 cells/well) were seeded into 96-well plates, incubated at 37 °C overnight, and pre-incubated with a 1:500 dilution of serum for 15 min at room temperature. Effector cells were subsequently applied per the manufacturer’s instructions. After 6 h of co-incubation, Bio-Glo™ Reagent was added, and luminescence was measured.

### 2.11. Antibody-Dependent Cell-Mediated Phagocytosis Assay

Antibody-dependent cell-mediated phagocytosis (ADCP) was measured against CT26-HER2 cells and CT26 cells using a FcgRIIa-H ADCP Reporter Bioassay (Promega, Madison, WI, USA, G9901). Target cells (5000 cells/well) were seeded into 96-well plates, incubated at 37 °C overnight, and pre-incubated with a 1:500 dilution of serum for 15 min at room temperature. Effector cells were subsequently applied per the manufacturer’s instructions. An effector to target ratio = 10:1 was tested. After 6 h of co-incubation, Bio-Glo™ Reagent was added, and luminescence was measured.

### 2.12. Complement-Dependent Cytotoxicity Assay

Complement-dependent cytotoxicity (CDC) was measured against CT26-HER2 cells and CT26 cells as follows: Briefly, target cells were seeded into 96-well plates (7500 cells/well), incubated at 37 °C overnight, and pre-incubated with 1:100-diluted serum from immunized mice at 37 °C for 1 h. After incubation, rabbit serum (1:100 dilution), as a source of complement, was applied and cultured at 37 °C for 2.5 h. Cytotoxicity was measured using the CytoTox 96 Nonradioactive Cytotoxicity Assay (Promega, Madison, WI, USA, G1780) to measure lactate dehydrogenase (LDH) release in the culture medium as evidence of cytotoxicity. Target cells incubated with a lysis buffer were used as a positive control, and target cells incubated with the medium were used as the negative control. The percentage of cytotoxicity was calculated.

### 2.13. In Vivo Studies

Animal procedures were performed in accordance with the guidelines of the Canadian Council on Animal Care. The animal ethics protocol was approved by the Biopharmaceutical Research Inc. (BRI) Animal Care Committee. SPF-grade female Balb/c mice were obtained from Envigo. Mice were quarantined for at least 7 days prior to being placed in the study. Mice were immunized subcutaneously with two doses of VG2062 (5 × 10^6^ PFU/mouse), or VG22401 (5 × 10^6^ PFU/mouse), or the vehicle. Two subcutaneous administrations were performed at two-week intervals.

For efficacy studies, 14 days after pre-immunization, bilateral CT26-HER2 (2.5 × 10^6^ cells/tumor) tumors were subcutaneously injected into the left and right flank. Once the tumor bump became visible, mice were injected intratumorally into the right flank with single doses of VG2062 (1 × 10^7^ PFU/mouse), or VG22401 (1 × 10^7^ PFU/mouse), or the vehicle. Tumor volumes were monitored at least twice a week and measured using a caliper (length × width × depth × 0.5236). The tumor growth inhibition percentage was calculated using the following formula: (TGI)% = [1 − (mean RTV (current tumor volume/tumor volume at day 0 post-intratumoral-treatment) of the treated group)/(mean RTV of the control group)] × 100 (%).

### 2.14. Statistical Analysis

For statistical analysis and data visualization, Microsoft^®^ Excel^®^ and GraphPad Prism version 9.4.1 were utilized. The Log-rank (Mantel–Cox) test, Tukey’s multiple comparison test, t-test, or ordinary two-way ANOVA with Dunn’s multiple comparisons test was employed to calculate *p*-values.

## 3. Results

### 3.1. Construction and Characterization of HER2-Expressing Virus

**Figure 1:**
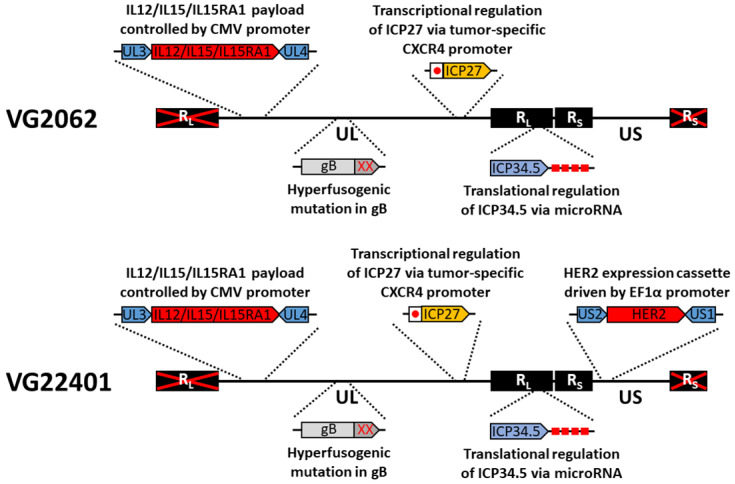
Schematic diagram of HER2-expressing virus.

Standard bacterial artificial chromosome recombineering techniques were used to construct HER2-expressing recombinant HSV-1 viruses based on HSV-1 strain 17 (Figure 1). The virus VG22401 incorporated a transcriptional and translational dual-regulated mechanism [25], and was further engineered to express a cytokine payload consisting of IL12, IL15, and the IL15 alpha receptor subunit isoform 1. VG22401 also included a truncated form of gB to enhance viral spread, and additionally encoded an expression cassette for the extracellular domain of HER2, while the backbone virus VG2062 lacked the HER2 expression cassette but was otherwise identical to VG22401.

### 3.2. HER2-Expressing HSV-1 Displays an Antitumor Effect in Human and Mouse Colorectal Cancer Cells

**Figure 2:**
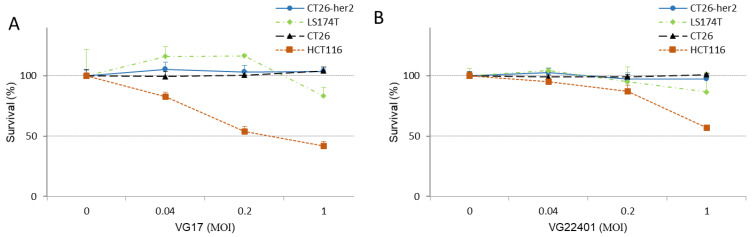
**Cytotoxic effect of VG22401 and VG17 in human and mouse cancer cells in vitro.** Cell survival percentages were determined using a Cell Counting KIT 8 assay to evaluate the cytotoxic effects of VG17 (**A**), and VG22401 (**B**) viruses in human cancer cell monolayers (HCT116 and LS174T) and mouse cancer cell monolayers (CT26 and CT26-HER2) at 72 h after infection with MOIs of 0.04, 0.2, and 1.

To evaluate the cytotoxic effects of the HER2-expressing virus VG22401, we conducted an in vitro study by exposing human and mouse colorectal cancer cells to either VG22401 or to the wild-type virus (VG17). Among the four types of tumor cells tested, human colorectal cell line HCT116 displayed the highest sensitivity to both viruses (Figure 2). However, mouse tumor cell lines (CT26 and CT26-HER2) exhibited lower levels of cytotoxicity.

### 3.3. Replication and Payload Expression of HER2-Expressing HSV-1 in Human and Mouse Colorectal Cancer Cells In Vitro

**Figure 3:**
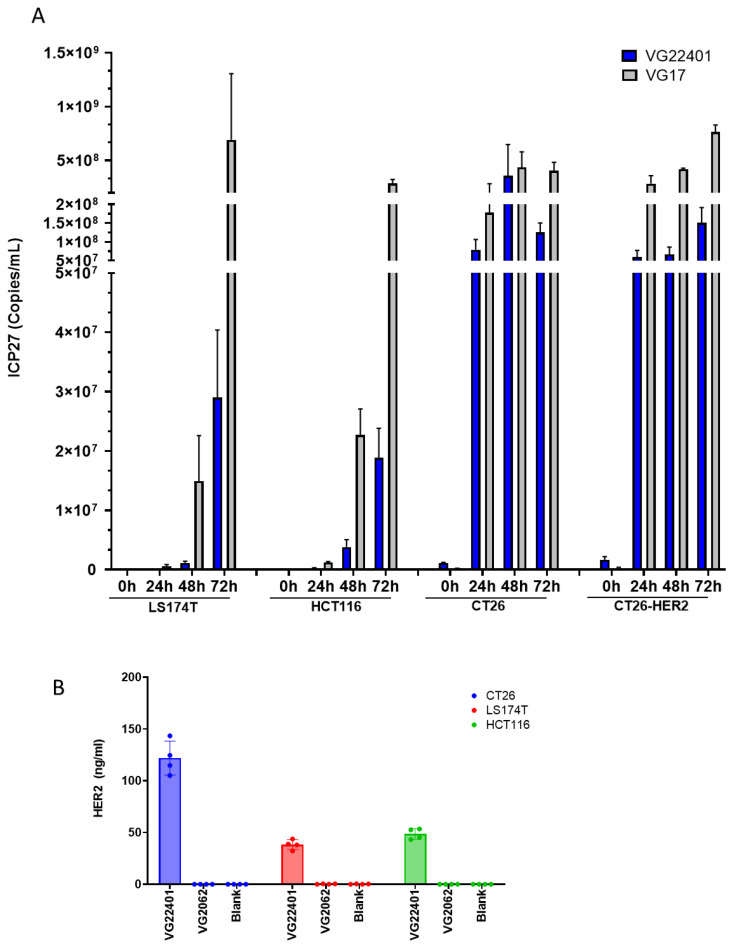
**Virus replication and payload expression in human and mouse colorectal cancer cells in vitro.** (**A**) Cells were infected with VG22401 or VG17 viruses. LS174T and HCT116 human colon cancer cells were infected at MOI = 0.001; CT26 and CT26-HER2 murine colon cancer cells were infected at MOI = 3. All infections were carried out in duplicate. Total DNA was extracted from the samples at the indicated times post-infection. To determine viral yield, ICP27 gene copy numbers were quantified by qPCR. (**B**) To determine HER2 payload expression, CT26, LS174T, and HCT116 cells were infected with HER2-expressing VG22401, or with the backbone virus VG2062. LS174T and HCT116 human colon cancer cells were infected at MOI = 3; CT26 murine colon cancer cells were infected at MOI = 6. Supernatants were collected 48 h post-infection. All infections were carried out in duplicate. Concentration of HER2 in the supernatant of the infected cells was analyzed by ELISA.

To evaluate viral replication capability, LS174T and HCT116 human colorectal cancer cells were infected with VG17 and VG22401 viruses. Among these viruses, the wild-type virus (VG17) showed the highest potency and replicated faster than the tumor-specific VG22401 virus that expresses HER2 (Figure 3A). To confirm efficient replication of the viruses in murine cells, we used CT26 and CT26-HER2 mouse colorectal cancer cells. The results showed that both VG17 and VG22401 viruses were able to replicate in both CT26 and CT26-HER2 cells (Figure 3A). To evaluate the level of HER2 payload expression in mouse and human colon cancer cell lines, LS174T and HCT116 human colorectal cancer cells were infected with VG2062 or VG22401 viruses. HER2 transgene expression was evident in VG22401-virus-infected cells in both mouse (CT26) and human (LS174T and HCT116) colorectal cancer cell lines (Figure 3B).

### 3.4. Immunization with HER2-Expressing oHSV-1 Generates Humoral and Cellular Anti-HER2 Immune Responses

**Figure 4:**
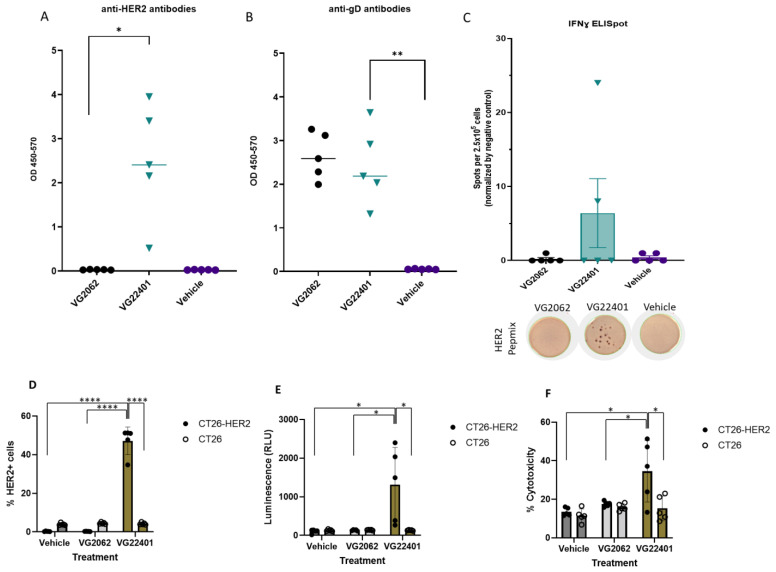
**Induction of humoral and cellular immune response by an HSV-1 expressing HER2.** Humoral immune response to HER2 (**A**) or oHSV-1 (**B**) after vaccination with VG22401 (black dots) and VG2062 (green triangles) viruses was evaluated by ELISA. Serum isolated from mice 35 days post-vaccination was diluted in series and incubated on plates pretreated with recombinant HER2 protein or with HSV-1 envelope glycoprotein D (gD). Anti-HER2 and anti-gD IgG antibody levels were measured with HRP-conjugated goat anti-mouse IgG and TMB, and absorbance at 450 nm was quantified (optical density). Plots show median values. An unpaired t-test was performed, and two-tailed *p*-values < 0.05 (*) and *p* < 0.002 (**) are shown. (**C**) Cellular immune response was measured by IFNγ ELISPOT assay with splenocytes isolated from mice. ELISPOT bar graph shows mean frequencies of IFNγ spots induced by HER2 peptide mix stimulation per 2.5 × 10^5^ splenocytes. Error bars indicate SEM. The bottom panel shows representative wells with IFNγ spots after stimulation with HER2 peptide mix. (**D**–**F**) Binding of serum anti-HER2 antibodies to HER2-expressing tumor cells. CT26-HER2 and HER2-negative parental CT26 cells were co-incubated with 1:100 dilution of serum followed by APC-conjugated, anti-mouse IgG antibody. The antibody binding to cell-surface-expressed HER2 was measured by flow cytometry (*, *p* < 0.05; ****, *p* < 0.0001) (**D**). Antibody-dependent cell-mediated cytotoxicity (ADCC) was measured against CT26-HER2 and CT26 cells using mFcgRIV ADCC Reporter Bioassay. Target cells were seeded, incubated at 37 °C overnight, and pre-incubated with 1:500 dilution of serum for 15 min at room temperature. Effector cells were subsequently applied per the manufacturer’s instructions. Effector to target ratio = 10:1 was tested. After 6 h of co-incubation, Bio-Glo™ Reagent was added, and luminescence was measured (*, *p* < 0.05) (**E**). Complement-dependent cytotoxicity (CDC) was assessed against CT26-HER2 and CT26 cells. Target cells were pre-incubated with 1:100 dilution of serum for 1 h at room temperature, and rabbit complement (1:100 dilution) was subsequently added for 2.5 h. Lactate dehydrogenase (LDH) released in the culture medium was measured and the percentage cytolysis was reported (*, *p* < 0.05) (**F**).

In order to assess the humoral and cellular immune responses to VG22401 and VG2062 viruses using a homologous prime–boost approach in immunocompetent BALB/c mice, the animals were subcutaneously immunized twice at 14-day intervals with 1 × 10^7^ PFU/mouse of VG22401 virus, VG2062 virus, or the vehicle control. On day 35 after the start of treatment, mice were euthanized, and their spleens and blood serum were collected and stored. ELISA was performed to measure levels of anti-HER2 and anti-gD antibodies in the collected blood serum samples. The gD protein is a ubiquitous immunogenic surface glycoprotein produced by the herpes simplex virus (HSV), and the VG22401 virus further encodes a HER2 expression cassette. Accordingly, anti-HER2 antibodies were only detected in mice that were immunized with VG22401 (Figure 4A), while anti-gD antibodies were found in mice treated with VG22401 and VG2062 (Figure 4B). HER2-PepMix-stimulated ELISPOT assays on the harvested splenocytes demonstrated an anti-HER2 cellular response in mice immunized with HER2-expressing virus (VG22401), while mice immunized with the VG2062 virus or with the vehicle control were negative (Figure 4C).

### 3.5. Mice Vaccinated with oHSV-1 Encoding HER2 Produce Serum Antibodies That Induce Cytotoxicity against HER2-Expressing Tumor Cells

We observed the binding of serum anti-HER2 antibodies to HER2-expressing tumor cells (CT26-HER2) using serum from VG22401-immunized mice. However, anti-HER2 antibodies did not bind to the parental HER2-negative CT26 cells and no serum anti-HER2 activity was observed in mice immunized with the vehicle or with the non-HER2-expressing HSV-1 VG2062 (Figure 4D). Antibody-dependent cell-mediated cytotoxicity (ADCC) assays showed that serum from mice immunized with HER2-expressing VG22401 virus successfully destroyed a large proportion of CT26-HER2 cells, while sparing HER2-negative parental CT26 cells (Figure 4E). Interestingly, complement-dependent cytotoxicity against CT26-HER2 cells was also observed (Figure 4F).

### 3.6. HER2 Payload Expression Enhances the Antitumor Effectiveness of oHSV-1 in a Bilateral Syngeneic HER2(+) Mouse Tumor Model

**Figure 5:**
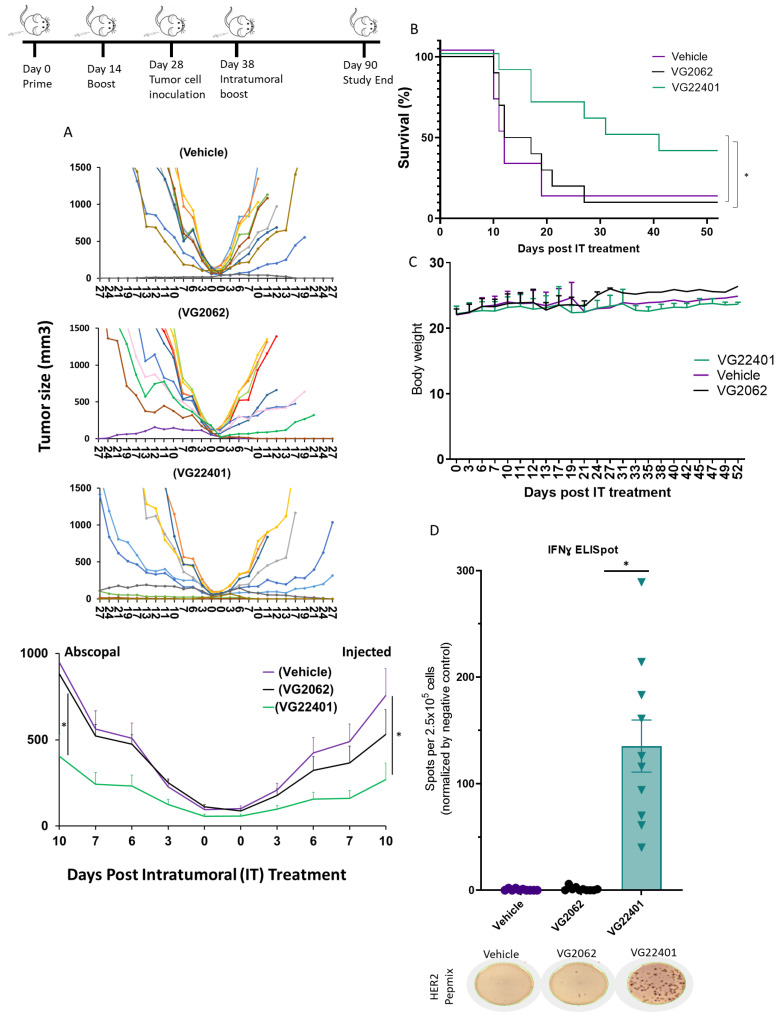
**HER2 payload improves the antitumor effect of oHSV-1 and induces a T cell response in a HER2-vaccinated bilateral, syngeneic murine tumor model.** Immunocompetent BALB/c mice were randomized into three groups and primed/boosted with VG2062 virus, VG22401 virus, or vehicle control. The mice were subsequently inoculated with CT26-HER2 tumors on both the left and right flanks. Once a tumor bump was visible, the tumors located on the right flank were injected intratumorally with a single dose of the same viruses used for immunization (1 × 10^7^ PFU/mouse), or with vehicle control. Tumor volumes were measured using a caliper, and the tumor growth curve illustrates the regression of the injected and abscopal tumors. The upper panel illustrates the tumor volume of individual animals, while the lower panel displays the average tumor volume (n = 10) for each group (**A**). The Kaplan–Meier survival curve was constructed using the time it took for mice tumors to grow to 1500 mm^3^ after intratumoral treatment (**B**), while the body weight change curve shows the post-treatment-changes in body weight (**C**). Cellular immune response was measured by IFNγ ELISPOT assay with splenocytes isolated from mice prime/boosted with VG2062 virus (black dots) and VG22401 virus (green triangles) when tumor size reached 1500 mm^3^. ELISPOT bar graph shows mean frequencies of IFNγ spots induced by HER2 peptide mix stimulation per 2.5 × 10^5^ splenocytes. Error bars indicate SEM. Ordinary two-way ANOVA, with Dunn’s multiple comparisons test, was performed to analyze the ELISPOT results. Two-tailed *p*-values are shown. The bottom panel shows representative wells with IFNγ spots after HER2 peptide mix stimulation (**D**). Asterisks (*) represent *p* values below 0.05.

To evaluate the efficacy of a homologous prime–boost HSV-1 vaccination strategy in immunocompetent mice bearing CT26 tumors expressing HER2, mice were randomly assigned to three groups and immunized twice at 14-day intervals with VG2062, VG22401, or the vehicle control. At 28 days post-treatment-initiation, mice were inoculated with CT26-HER2 cells on both the right and left flanks to generate an abscopal tumor model. At 38 days post-treatment-initiation, tumors on the right flank were injected intratumorally with the same virus used for pre-immunization, and tumor volumes were measured until euthanasia. Tumor growth curves indicated that mice subcutaneously primed and intratumorally boosted with HER2-expressing virus (VG22401) had better antitumor efficacy compared to mice treated with the vehicle and non-HER2-expressing virus (VG2062). A total of 4 out of 10 of the mice treated with VG22401 achieved a complete response (CR) on the injected side (Figure 5A) and also showed a survival benefit (Figure 5B), while only 2 out of 10 mice treated with non-HER2-expressing VG2062 virus showed CR on the injected side. The average body weight of all mouse groups remained stable during the study (Figure 5C).

We further examined the effect of each therapeutic regimen on immunological modulation, particularly the induction of anti-HER2 T cell responses. Thus, when the mice reached an endpoint due to tumor burden, we collected their spleens to assess the T cell response induced by different treatment groups. IFNγ ELISPOT assay revealed that mice treated with VG22401 showed an increase in IFNγ production after being stimulated with HER2 PepMix, when compared to splenocytes from mice treated with VG2062 or the vehicle control (Figure 5D).

## 4. Discussion

HER2, a transmembrane glycoprotein in the EGF superfamily, is widely expressed in epithelial cells and over-expressed in a large number of breast carcinomas. Pre-existing anti-HER2 immunity levels are typically too low to trigger an apparent therapeutic effect. Consequently, vaccines designed to target HER2-related antigens must overcome pre-existing immune tolerance to stimulate a potent and long-lasting immune response.

HER2-targeted antibodies, such as trastuzumab and pertuzumab, have become the standard treatment for HER2-positive breast cancer. The discovery of trastuzumab nearly 25 years ago marked a significant advancement in the management and development of drugs for HER2+ breast cancer [30]. Despite their effectiveness in disease control and survival improvement, anti-HER2 therapies carry potential adverse events. Notably, trastuzumab is associated with cardiac dysfunction, especially when combined with anthracyclines [30,31]. However, dual HER2-targeted therapy using pertuzumab and trastuzumab has not been demonstrated to worsen cardiotoxicity [32]. Conversely, the use of HER2 vaccines is generally well-tolerated and is associated with a low absolute risk of mortality or treatment discontinuation due to therapy-related adverse effects [33]. Interestingly, combining trastuzumab with a HER2/neu vaccine is associated with minimal toxicity [34]. Although some breast cancer vaccines were well-tolerated and able to generate detectable immune responses in initial trials, none of them exhibited significant clinical benefits in subsequent phase 3 trials [15]. The development of oncolytic viruses (OVs) as a cancer therapy has shown great promise in recent years, with several OVs being evaluated in clinical trials for a range of cancer types [35]. Despite the potential, OV therapy still faces several challenges, including limited efficacy in advanced cancers and immune evasion by tumor cells [36]. In this study, we sought to explore other immune regulatory factors that could better arm oHSV-1 to improve its antitumor efficacy.

In our previous study, we found that pre-existing anti-HSV immunity could enhance the oHSV-1-induced antitumor effect when the virus was intratumorally administrated [37]. In the present study, the mice were not only vaccinated with HSV-1 but also with HER2. This was carried out by administering HER2-expressing oHSV-1 subcutaneously to elicit not only anti-HSV-1 but also anti-HER2 immunity. Our findings validate the generation of both anti-HSV-1 and anti-HER2 antibodies. Furthermore, the anti-HER2 antibody initiates the eradication of tumor cells via ADCC and CDC mechanisms. As we hypothesized, priming with HER2/HSV-1 followed by boosting with an intratumoral injection of the same HER2-expressing oHSV-1 vector shows stronger efficacy when compared to a similar prime–boost strategy using non-HER2-expressing oHSV-1.

Infection with oHSV-1 initiates a prompt activation of innate immune responses in the host [38]. Mice primed and boosted with HER2-expressing oHSV-1 also generated an anti-HER2 systemic cellular response, resulting in enhanced tumor regression compared to mice primed and boosted with HER2 non-expressing oHSV-1. These data illustrate that immunization against HER2 may influence the infiltration of HER2-specific T cells into the tumor mass after intratumoral boost with the same virus, which leads to the further killing of tumor cells. Vaccine-induced HER2-specific CD8+ T cells are essential for generating an antitumor response. These activated T cells can infiltrate the tumor microenvironment and directly kill the cancer cells or trigger the immune system to mount an effective defense against tumor proliferation [39,40]. Furthermore, compared to using a HER2 vaccine alone, an intratumoral oHSV-1 booster alters the tumor microenvironment by attracting innate immune cells as well as HER2-specific T cells, leading to an enhanced antitumor effect. In addition, it appears that intratumoral boosting with VG22401 also enhances systemic antitumor effects. This is likely due to an altered tumor microenvironment triggered by OV-mediated cell lysis, which further releases other tumor antigens to initiate systemic immune responses.

However, this study does not provide information about the effectiveness of prime and boost with HER2-expressing oHSV-1 in inducing an antitumor response against HER2(+) tumor cells in individuals who have previously been exposed to HSV-1. It is reasonable to assume that individuals who have recently contracted HSV-1 may possess a strong anti-HSV-1 immune response and may not be suitable candidates for this treatment approach. A previous study involving a murine model of ocular HSV infection demonstrated that HSV reinfection with an autologous strain can occur within 30 days of the primary infection. Likewise, our preliminary findings in mouse models suggest that a prime and intratumoral boost interval of less than 5–6 weeks may diminish the effectiveness of the intratumoral oncolytic activity of oHSV-1. Nonetheless, reinfection with exogenous HSV-1 may happen [41,42], and a prior study has shown that inoculation of HSV-1 can produce exogenous reinfection in patients with frequent and recurrent herpes simplex infection [43]. This evidence supports the idea that regardless of HSV-1 seropositivity status, the prime and boost approach with HER2-expressing oHSV-1 should remain effective unless the initial priming occurs during the very early stages of HSV-1 exposure. Future research should study the extent of the neutralizing anti-HSV-1 effects and assess this prime–boost approach in animal models that have been pre-immunized against HSV-1.

## 5. Conclusions

Our findings demonstrate that combining pre-immunization by HER2-expressing oHSV-1 with intratumoral oHSV-1 therapy triggers a robust antitumor humoral and cellular response in HER2(+) tumor models, leading to significant improvements in both local and systemic tumor control. Taken together, the results suggest that intratumorally injected oncolytic viruses overexpressing a tumor-associated antigen can significantly boost OV-mediated antitumor immunity and efficacy after priming with a peripherally delivered tumor vaccine.

## Data Availability

Raw data is available upon request.

## Supplementary Materials

The following supporting information can be downloaded at: https://www.mdpi.com/article/10.3390/vaccines11121805/s1, Figure S1: Generation of a HER2-expressing mouse tumor model, Figure S2: Administration of HER2-expressing oHSV-1 directly into a tumor shows an improved antitumor response when preceded by a priming dose of HER2-expressing oHSV-1 delivered subcutaneously, Figure S3: Biodistribution of CXCR4 driven TTDR virus (VG185LF) in BXPC3 tumor bearing mice model.
