# Supplementary material for: Prophylactic Vaccination and Intratumoral Boost with HER2-Expressing Oncolytic Herpes Simplex Virus Induces Robust and Persistent Immune Response against HER2-Positive Tumor Cells
Source: Vaccines (Basel). 2023 Dec 2;11(12):1805. doi: 10.3390/vaccines11121805 (PMC10747554; doi:10.3390/vaccines11121805)
Supplement: Supplementary file 1 [file vaccines-11-01805-s001.zip › vaccines-2660161-supplementary.pdf]

SUPPLEMENTARY INFORMATION:

Figure S1: Generation of a HER2-expressing mouse tumor model

Multiple clones were tested by flow cytometry for the expression of erbB-2 using antibody Anti-ErbB2/c-Neu (Ab-5), clone TA-1. Clone 24 was selected based on the expression of erbB-2, as well as the ability to establish tumors in BALB/c mice. To develop the human HER2-expressing CT26 immunocompetent mouse model, we tested different numbers of CT26-HER2 tumor cells inoculated into BALB/c mice. Better tumor intake and growth were observed in mice inoculated with 2.5x10^6 and 5x10^6 cells.

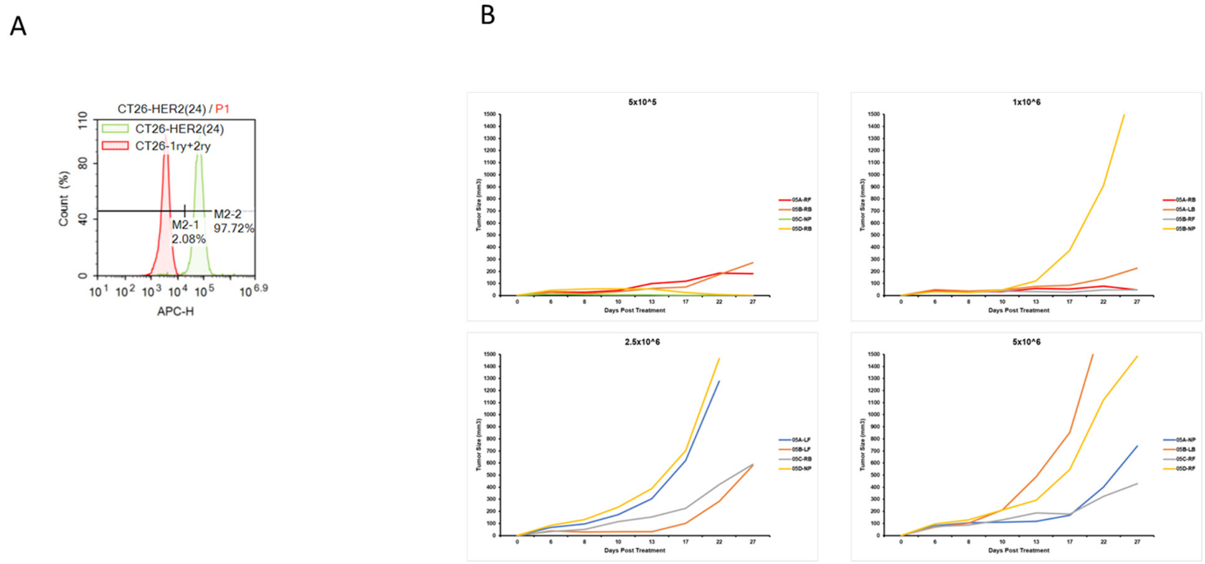

HER2 tumor development in BALB/c mice. A, CT26-HER2 clone #24 stained using anti-ErbB2/c-Neu. B, HER2-expressing CT26 tumor growth rate in BALB/c mice.

Figure S2: Administration of HER2-expressing oHSV-1 directly into a tumor shows an improved antitumor response when preceded by a priming dose of HER2-expressing oHSV-1 delivered subcutaneously

Recombinant virus construction: The structure of VG2044 was more similar to our previously published oncolytic HSV-1 VG161 (Chouljenko et al., 2020), which employed deletion of both ICP34.5 copies to ensure patient safety and facilitate virus replication in tumor cells. VG2044 was engineered to express a payload cassette consisting of IL12, IL15 and the IL15 alpha receptor subunit isoform 1, with each element separated by 2A peptides. The cytokine payload was inserted between viral genes UL3 and UL4, and its expression was controlled by a cytomegalovirus (CMV) promoter. VG2044 was further encoded an expression cassette for the extracellular domain of HER2 driven by the EF1a promoter, which was inserted between the HSV-1 genes US1 and US2.

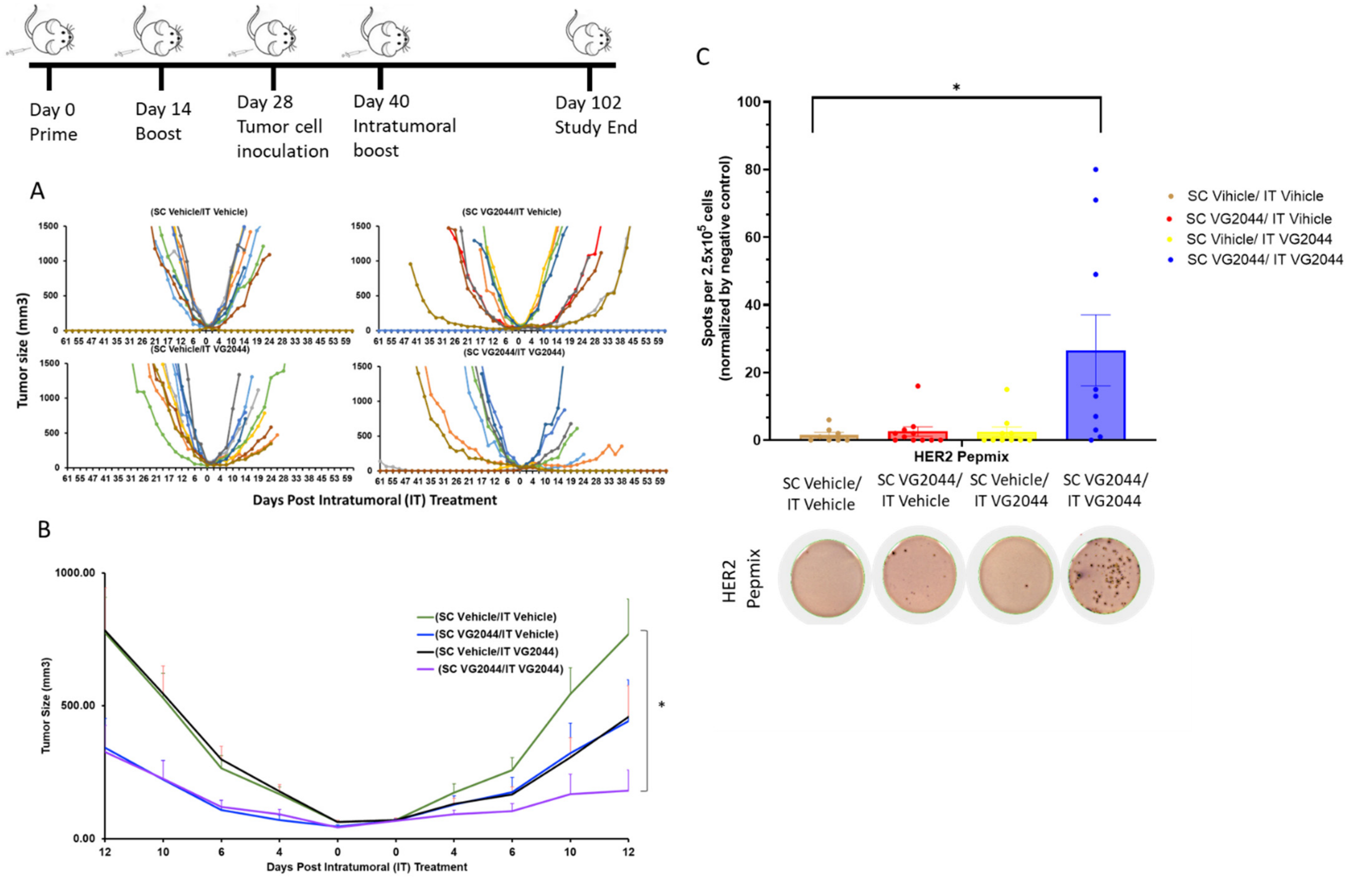

Prime and boost with HER2-expressing oHSV-1 increases the systemic antitumor effect *in vivo*. Immunocompetent BALB/c mice were randomized into groups and vaccinated through two subcutaneous (SC) administrations each time with 5x10^6 PFU/mouse of VG2044 virus or with vehicle control. The mice were subsequently inoculated with CT26-HER2 tumors on both the left and right flanks. Once tumor growth was visible, the tumors located on the right flank were injected intratumorally (IT) with a single dose of the same virus (1 x 10^7 PFU/mouse), or with control article. Tumor volumes were measured using a caliper, and the tumor growth curve illustrates the regression of the injected and abscopal tumors of individual mice (A) and the average of all mice in each group (B). Cellular immune response to the combination of vaccination and subsequent treatment with VG2044 was analyzed by IFNg ELISpot. Splenocytes of the treated mice were isolated and plated at 2.5×10^5 cells per well in duplicate and incubated with HER2 peptide mix (0.5μg per 10^6 cells) for 24 hours, (C). The number of spots in the stimulated samples were normalized by non treated control. * indicates a statistically significant difference between groups.

To investigate whether pre-existing HER2 immunity enhances the antitumor efficacy of HER2-expressing HSV-1, immunocompetent BALB/c mice were subcutaneously immunized twice at 14-day intervals with vehicle control, or with 5x10^6 PFU/dose of HER2-expressing VG2044 virus o. Subsequently, bilateral CT26-HER2 tumors were subcutaneously inoculated into both flanks of each mouse. Once the tumors had formed, tumors located in the right flank were intratumorally injected once with either vehicle control or virus (1x10^7 PFU/mouse). Among all treatments, mice that were vaccinated with VG2044 and then intratumorally injected with VG2044 showed the best antitumor efficacy and a pronounced abscopal effect, with 4 out of 10 animals achieving a complete response on the injected side and 3 out of 10 animals showing a complete response on the non-injected side (Figure S2-A). Average tumor size (Figure S2-B) also demonstrated that pre-immunization with VG2044 provides better tumor regression and tumor growth inhibition.

We further examined the effect of each therapeutic regimen on immunological modulation, particularly the induction of anti-HER2 T cell responses. Thus, when the mice reached an endpoint due to tumor burden, we collected their spleens to assess the T cell response induced by different treatment groups. IFN-g ELISPOT assay revealed that mice treated with VG2044 pre-immunization and intratumoral boosting exhibited the most significant HER2-specific T-cell responses compared to mice subjected to only pre-

immunization or intratumoral administration. Conversely, mice treated with vehicle or only intratumoral VG2044 or only pre-immunized with VG2044 virus failed to elicit any notable HER2-specific response (Figure S2-C).

Figure S3: Biodistribution of CXCR4 driven TTDR virus (VG185LF) in BXPC3 tumor bearing mice model.

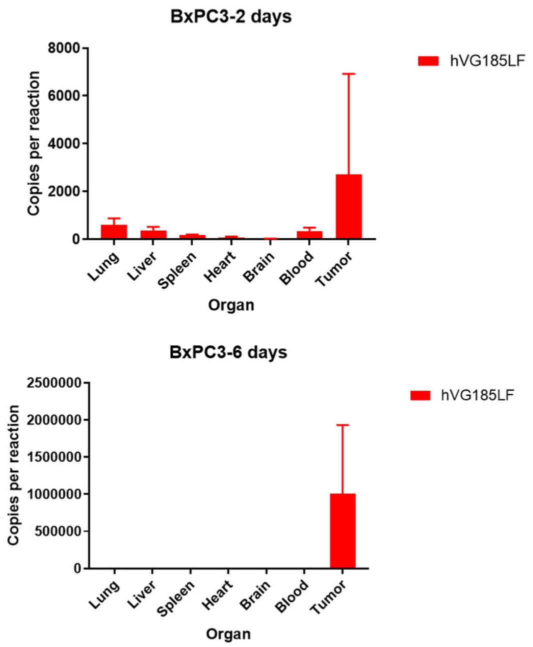

Subcutaneously, BxPC3 tumor-bearing mice (N=4) were treated intravenously (IV) with a CxCR4 promoter-driven oncolytic HSV-1 (hVG185LF) at a single dose of 5x10^7 PFU per mouse. Mice were harvested at day 2 and day 6 post-treatment, and tumor, lung, liver, heart, brain, spleen, and blood samples were collected. DNA was extracted from the collected samples for q-PCR. The bars represent the HSV-1 copies.
